# Screening for resistance against *Pseudomonas syringae* in rice-FOX *Arabidopsis* lines identified a putative receptor-like cytoplasmic kinase gene that confers resistance to major bacterial and fungal pathogens in *Arabidopsis* and rice

**DOI:** 10.1111/j.1467-7652.2010.00568.x

**Published:** 2011-05

**Authors:** Joseph G Dubouzet, Satoru Maeda, Shoji Sugano, Miki Ohtake, Nagao Hayashi, Takanari Ichikawa, Youichi Kondou, Hirofumi Kuroda, Yoko Horii, Minami Matsui, Kenji Oda, Hirohiko Hirochika, Hiroshi Takatsuji, Masaki Mori

**Affiliations:** 1 National Institute of Agrobiological SciencesTsukuba, Japan; 2 RIKEN, Plant Science CenterYokohama, Japan; 3 Research Institute for Biological Sciences, Okayama Prefectural Technology Center for Agriculture, Forestry, and FisheriesOkayama, Japan

**Keywords:** *Arabidopsis*, disease resistance, FOX hunting system, *Pseudomonas syringae*, receptor-like cytoplasmic kinase, rice

## Abstract

Approximately 20 000 of the rice-FOX *Arabidopsis* transgenic lines, which overexpress 13 000 rice full-length cDNAs at random in *Arabidopsis*, were screened for bacterial disease resistance by dip inoculation with *Pseudomonas syringae* pv. *tomato* DC3000 (*Pst* DC3000). The identities of the overexpressed genes were determined in 72 lines that showed consistent resistance after three independent screens. *Pst* DC3000 resistance was verified for 19 genes by characterizing other independent *Arabidopsis* lines for the same genes in the original rice-FOX hunting population or obtained by reintroducing the genes into ecotype Columbia by floral dip transformation. Thirteen lines of these 72 selections were also resistant to the fungal pathogen *Colletotrichum higginsianum*. Eight genes that conferred resistance to *Pst* DC3000 in *Arabidopsis* have been introduced into rice for overexpression, and transformants were evaluated for resistance to the rice bacterial pathogen, *Xanthomonas oryzae* pv. *oryzae*. One of the transgenic rice lines was highly resistant to *Xanthomonas oryzae* pv. *oryzae*. Interestingly, this line also showed remarkably high resistance to *Magnaporthe grisea*, the fungal pathogen causing rice blast, which is the most devastating rice disease in many countries. The causal rice gene, encoding a putative receptor-like cytoplasmic kinase, was therefore designated as *BROAD-SPECTRUM RESISTANCE 1.* Our results demonstrate the utility of the rice-FOX *Arabidopsis* lines as a tool for the identification of genes involved in plant defence and suggest the presence of a defence mechanism common between monocots and dicots.

## Introduction

The DNA sequences of entire genomes have already been determined in several organisms but comparatively little is known about the function of most genes. Functional genomics aims at determining the function of genes and, consequently, revealing their potential for future manipulation and use. Functions of many plant genes have been discovered in basic researches involving the model dicot, *Arabidopsis*. A number of gene functions have also been identified in rice, the model plant for monocots; however, most of the rice genes still remain functionally uncharacterized, and little experimental evidence is available to support the putative functions ascribed in genomic annotations.

Insertional mutagenesis, using transfer DNA (T-DNA) or transposable elements, offers a direct approach towards determining gene functions. In this method, gene activity is either disrupted or activated by mutator elements that also function as tags for subsequent sequence identification (Hirochika *et al.*, 2004; Krishnan *et al.*, 2009). However, the preferential insertion of the mutator elements into specific chromosomal segments of the target genome will lead to an arguably limited coverage of genes in the target genome (Hsing *et al.*, 2007). A more important limitation of the gene activation approach is that gene activation can occur up to 12 kb upstream and downstream regions of the insertion site and this might lead to complex phenotypes because of the upregulation of neighbouring genes (Ichikawa *et al.*, 2003). Furthermore, insertion of an activator cassette into a gene, especially into a coding region, can lead to gene disruption or other unexpected outcomes.

Specific gain-of-function mutants can be produced by introducing target genes under the control of a constitutive promoter and a suitable terminator sequence into a host genome. Constitutive expression (or overexpression) of these genes often results in distinct phenotypes that can be used to deduce their corresponding functions. Recently, Ichikawa *et al.* (2006) reported the development of a new tool for functional genomics of *Arabidopsis* cDNA, Full-length cDNA OvereXpressor gene hunting (FOX hunting) system, wherein 10 000 full-length *Arabidopsis* cDNAs were inserted downstream to a CaMV promoter. They generated about 15 000 transgenic *Arabidopsis* lines that expressed *Arabidopsis* full-length cDNAs via the *Agrobacterium*-mediated floral dip transformation method. They found about 1500 morphological mutants and identified some causal genes (Ichikawa *et al.*, 2006). In a subsequent report, Fujita *et al.* (2007) made a mini-scale FOX line for a pool of transcription factors to characterize their possible roles in salt tolerance of plants.

In rice, Nakamura *et al.* (2007) reported on the generation of a population of rice transgenic lines overexpressing 13 980 independent full-length rice cDNAs under the control of the maize *Ubiquitin-1* promoter. Similarly, a rice-FOX *Arabidopsis* population of 23 000 lines was developed by introducing 13 000 full-length rice cDNAs under the control of CaMV *35S* promoter into *Arabidopsis* ecotype Columbia (Kondou *et al.*, 2009). By using these rice-FOX *Arabidopsis* lines, several rice genes were identified that are involved in heat tolerance, salt tolerance and nitrogen metabolism (Yokotani *et al.*, 2008, 2009a,b; Albinsky *et al.*, 2010).

Plant diseases are one of the major limiting factors in crop production. Utilizing genes involved in plant defence mechanisms is an approach to develop disease-resistant crops. Resistance (*R*)-gene-mediated resistance has been widely used in breeding; however, the resistance is limited to specific races of pathogens and often breaks down because of the outgrowth of mutated pathogens after a few years of commercial cultivation in the field (Bonman *et al.*, 1992). Thus, disease resistance that is durable and effective against broad spectrum of pathogens (or pathogen races) is of invaluable agronomical importance (Kou and Wang, 2010). Therefore, identifying new genes that can confer such disease resistance traits to crops is among the subjects of top priority in plant science.

In this study, we screened the rice-FOX *Arabidopsis* lines for resistance to *Pseudomonas syringae* pv. *tomato* DC3000 (*Pst* DC3000), the major bacterial pathogen of *Arabidopsis,* with the aim of rapidly identifying defence-related genes of rice in a heterologous plant screening system. This screening successfully identified many rice genes that conferred resistance to *Pst* DC3000 and some of them also conferred resistance to the fungal pathogen *Colletotrichum higginsianum* in *Arabidopsis*. When overexpressed in rice, one of these conferred strong resistance to both *Xanthomonas oryzae* pv. *oryzae* (*Xoo*) and *Magnaporthe grisea*, two of the most devastating rice diseases. Thus, our screening identified a possibly important gene that can confer resistance to both bacterial and fungal pathogens in both monocots and dicots.

## Results

### Screening for resistance to *Pst* DC3000

**Figure 1:**
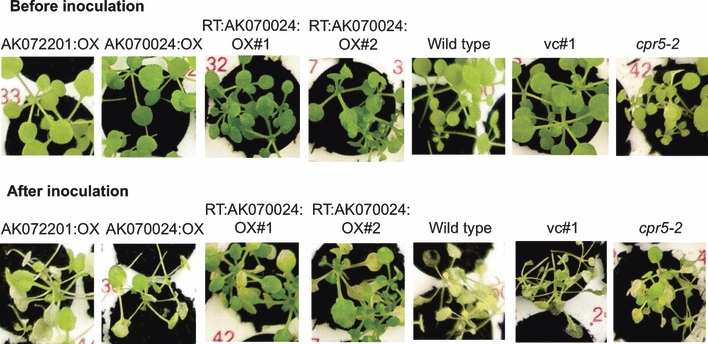
Phenotypic responses to *Pst* DC3000 dip inoculation. Upper panel shows 3-week-old T_2_ plants used for *Pst* DC3000 inoculation. Lower panel shows typical disease symptoms 6 days after inoculation with 10^8^ cfu/mL of *Pst* DC3000. The wild-type (Col-0) and vector control (VC#1) plants died, but the AK072201:OX, AK070024:OX and *cpr5-2* (resistance mutant to *Pst* DC3000) plants survived. RT:AK070024:OX#1 and RT:AK070024:OX#2 also survived the *Pst* DC3000 screen. RT:AK070024:OX#1 and RT:AK070024:OX#2 are independent retransformed lines for AK070024.

Conventional protocols for screening and evaluation of disease resistance traits (see Fig. S1a) are difficult to apply to a large population of plants. In conventional methods that use plants grown in non-aseptic conditions, the plant's responses to pathogen inoculation can be modulated by abiotic factors like humidity and ventilation, and biotic factors like insects and microbes. The elimination of those extraneous factors is an essential requirement to attain repeatability and reliability in the screening of large populations. Therefore, we developed a new system to avoid these problems (Fig. S1b). *Arabidopsis* plants were grown in an aseptic condition free from drought and pest infestation. They were dip inoculated with bacteria for 30 s and this was their only exposure to non-sterile conditions. The inoculated plants usually turned yellow after the 3-day incubation in the dark, but control resistant plants *cpr5-2* (Boch *et al.*, 1998), in which salicylic acid (SA) signalling pathway is constitutively activated, recovered its green colour presumably as a result of de novo chlorophyll synthesis. Hence, the ability to recover from the chlorosis induced by *Pseudomonas* and generate healthy green tissue after stringent inoculation and incubation was deemed as an indicator of plant resistance. This protocol was used to screen 20 000 FOX lines for resistance to *Pst* DC3000 to discover novel genes involved in rice defence mechanism to pathogens. Typical examples of the FOX lines that were resistant to *Pst* DC3000 are shown in Fig. 1. Three-week-old T_2_ plants (upper photographs) were inoculated with 10^8^ cfu/mL of *Pst* DC3000, and disease symptoms were evaluated 6 days after inoculation (lower photographs). Wild-type (Col-0) and vector control (VC#1) plants were apparently killed by the screening method. In contrast, the plants from some FOX lines (AK072201:OX, AK070024:OX) showed many green healthy leaves 6 days after inoculation, similar to the resistant control plants, *cpr5-2*. Two independent retransformed lines for AK070024, RT:AK070024:OX#1 and RT:AK070024:OX#2, also showed phenotypes similar to *cpr5-2*.

This high-throughput screening enabled the evaluation of 20 000 transgenic lines for resistance to *Pst* DC3000. Screening at inoculum levels (0.5–2 × 10^8^ cfu/mL) led to the isolation of 1620 lines in the first screening and 204 lines in the second screening (replicated twice each time). The third screening, at inoculum levels of 1–2 × 10^8^ cfu/mL, resulted in the selection of 72 lines (Table 1).

**Table 1:** Number of rice-FOX *Arabidopsis* lines that survived dip inoculation with *Pst* DC3000

	First screening	Second screening	Third screening
Inoculum level (cfu/mL)	0.5–2 × 10^8^	0.5–2 × 10^8^	1–2 × 10^8^
Screened	20 000	1620	204
Resistant*	1620	204	72†
% Resistant lines	8.1	1.0	0.36

* Lines that survived 6 days after dipping in inoculum containing *Pst* DC3000.

† 59 lines with unique gene inserts plus 13 independently transformed lines with gene inserts identical to one of the 59 lines.

### Validation of the high-throughput screening procedure by bacterial count

**Figure 2:**
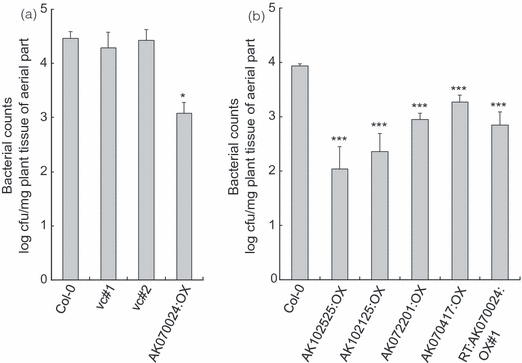
Bacterial growth in plants. Five-week-old *Arabidopsis plants* were inoculated with *Pst* DC3000 by dipping in a bacterial suspension (10^6^ cfu/mL), and the numbers of bacteria in the aerial part of the plants were counted after 3 days. Bars indicate the SD (*n* = 4). (a) The differences in bacterial count between Col-0 (or vector controls) and AK070024:OX plants are significant at 5% (*) by *t*-test. Col-0, wild type; vc, vector control. (b) The differences in bacterial counts in plants between Col-0 and the selected *Pst* DC3000-resistant transgenic *Arabidopsis* lines are significant at 5% (*) or 0.1% (***), respectively. RT:AK070024:OX#1 is the same retransformed line as that used in Fig. 1.

Our binomial (survive or die) screening protocol identified FOX lines that survived three independent dip inoculations with *Pst* DC3000 at relatively high inoculum levels under conditions that favoured successful infection. As our selection was based on the reinitiation of de novo chlorophyll production in erstwhile chlorotic leaves, it can be argued that the observed ‘resistance’ was because of factors other than suppression of bacterial growth. Hence, we also counted bacteria numbers in inoculated plant tissues of a few selected lines to examine whether the plant survival was because of the repression of the bacterial population. We counted the bacteria according to the protocol of Katagiri *et al.* (2002). As expected, the bacterial counts (in colony-forming units/mg plant tissue) in two vector control lines were similar to that in the wild type, Col-0 (Fig. 2a). The bacterial count in the resistant FOX line AK070024:OX was significantly lower than those in the wild type and vector control (Fig. 2a). In another experiment, the bacterial counts in four randomly selected resistant lines were significantly lower as well (Fig. 2b). In particular, the bacterial counts in AK102525:OX plants were about 1/100 of that in wild type. This protocol was also applied to one of retransformed plant (RT:AK070024:OX#1), which also showed significantly lower bacterial count compared to the wild type (Fig. 2b).

### FOX lines with single rice cDNA insert showing resistance to *Pst* DC3000

The *Pst* DC3000-resistant phenotype observed in T_2_ population could be because of gene disruption or other unexpected mutations that may have occurred during the *Agrobacterium*-mediated transformation process. Hence, it is important to verify the phenotypes in independent transformant lines overexpressing the same cDNA, either by finding these in the existing T_2_ population of the FOX lines or by retransformation. With this criterion, 19 single-insert rice cDNAs have so far been confirmed to enable the survival of transgenic *Arabidopsis* after exposure to our screening protocol (Table 2). These cDNAs conferred *Pst* DC3000 resistance to the corresponding transgenic lines in at least five independent screens, i.e., in three screens with the original FOX population and at least two screens with retransformed lines. qPCR verified the overexpression of insert cDNAs in all of the randomly selected five lines of the 19 original screened lines (data not shown). Another group of 16 lines that consistently showed high resistance to *Pst* DC3000 still need verification through repeat screening of independently transformed lines (Table 2). We currently refer to these 35 genes as *RPD* (*Resistance to PstDC3000*) genes.

**Table 2:** *Pst* DC3000 resistant lines with one cDNA insert

						Sequence in comparison with genomic DNA§		Response to		
								
Line No	Original line	Independently transformed line(s)*	Accession no.†	RAP ID‡	RAP description‡	cDNA	Protein	*C. higginsianum*¶	Xoo**	*Pst* counts††
*Resistance confirmed in multiple independent line(s) (19 lines)*										
1	K00714	RT	AK068846	Os01t0127300-01	SufBD family protein	Ok	Ok	S	S	
2	K21617	RT	AK103699	Os10t0530900-01	Similar to Glutathione S-transferase GST 30	1 ins	Ok	R	S	
3	K00841	RT	AK072201	Os01t0503400-04	Similar to metal transporter Nramp6	1 del	62% shorter	R	S	Yes
4	K15424	R06015, RT	AK070024	Os09t0533600-01	Similar to Avr9/Cf-9 induced kinase 1	Ok	Ok	R	R	Yes
5	K04135	K20450, RT	AK100547	Os02t0145600-01	Conserved hypothetical protein	Ok	Ok	S	S	
6	K25904	K18218(2inserts)	AK072899	Os09t0363900-01	Similar to HOTHEAD protein precursor	Ok	Ok	R	S	
7	K02342	K23019	AK102525	Os12t0619000-01	IQ calmodulin-binding region domain containing protein	Ok	Ok	R	S	Yes
8	K21204	K17730	AK102125	Os08t0250700-01	Thioredoxin domain 2 containing protein	Ok	Ok	S		Yes
9	K29409	RT	AK099032	Os03t0240500-01	Similar to Toc34-2 protein	Ok	Ok	S		
10	R04214	RT	AK069592	Os01t0232100-01	Similar to Tonoplast membrane integral protein ZmTIP4-3	1 ins	Ok	S		
11	K03301	RT	AK070417	Os03t0197100-01	Similar to Sugar transporter protein	Ok	Ok	S		Yes
12	K03216	K18912	AK101795	Os04t0382300-01	Similar to SNF1-related protein kinase regulatory gamma subunit 1	Ok	Ok	R		
13	K37838	K25231	AK070720	Os03t0563300-03	Similar to Mg-chelatase subunit (Fragment)	1 bs	Ok	S		
14	R05946	K02535(2inserts)	AK099542	Os06t0639550-01	Non-protein coding transcript	Ok	no protein	S		
15	R06202	K04148	AK071866	Os03t0738300-01	Hypothetical protein	1 bs	Ok	S		
16	R06736	K13623	AK101242	Os04t0346800-02	EAR repeat containing protein	1 ins	2× longer	S		
17	R06848	K13001	AK073641	Os06t0623600-01	Similar to Cinnamoyl-CoA reductase	Ok	Ok	S		
18	R03132	K19414, K30128	AK069465	Os07t0622100-01	Similar to Ribosomal protein s6 RPS6-2	1 bs	Ok	S		
19	K09018	RT	AK071510	Os06t0556200-01	Similar to Amino acid permease I	Ok	Ok	S		
*No independent lines available for confirmatory screening (16 lines)*										
20	K17110		AK101316	Os07t0435100-01	Similar to 26S proteasome subunit RPN12	1 del 2 bs	Ok	R		
21	K19720		AK072674	Os03t0333300-02	Similar to eukaryotic translation initiation factor 2 beta subunit	2 ins	40% longer	R	S	
22	K08435		AK068205	Os06t0661600-01	Zinc finger, DPH-type domain containing protein.	1 bs	Ok	S		
23	K17109		AK111889	Os10t0160000-01	Similar to Ubiquitin carboxyl-terminal hydrolase 12	Ok	Ok	S		
24	K04020		AK066139	Os09t0461700-01	Alpha/beta hydrolase fold-3 domain containing protein	1 bs	1 ac	R		
25	K37931		AK071286	Os01t0803300-01	Protein of unknown function DUF6	1 bs	1 ac	S		
26	K39531		AK099196	Os02t0590400-02	Lecithin:cholesterol acyltransferase family protein.	Ok	Ok	R		
27	K40223		AK065007	Os01t0978100-01	Similar to Cysteine synthase, mitochondrial precursor	Ok	Ok	S		
28	K40946		AK103235	Os02t0829100-01	Replication protein A 30 kDa	Ok	Ok	S		
29	R04016		AK102402	Os02t0489400-01	Similar to 40S ribosomal protein S8	2 bs	1 ac	S		
30	R06007		AK103707	Os01t0160800-01	Similar to Protein synthesis inhibitor II (Ribosome-inactivating protein II)	1 bs	Ok	S		
31	R05018		AK111775	Os01t0313300-01	Similar to EREBP-3 protein (Fragment)	Ok	Ok	R		
32	K03221		AK070873	Os04t0103100-01	Glycosyl transferase, family 43 protein	2 ins 1 del 2 bs	8.4% shorter	S		
33	K17538		AK070457	Os10t0190900-01	Multi antimicrobial extrusion protein MatE family protein.	1 del 1 bs	1 ac	S		
34	K31418		AK101216	Os10t0573900-01	NMD3 family protein.	1 del 2 bs	37% shorter	S		
35	K30521		AK073206	Os10t0573900-03	Similar to Nonsense mediated mRNA decay protein 3.	2 del 1 bs	Ok	S		

* RT represents retransformed.

† Accession No. provided by KOME (http://cdna01.dna.affrc.go.jp/cDNA/).

‡ ID and predicted protein annotation provided by RAP-DB (http://rapdb.dna.affrc.go.jp/).

§ KOME cDNA sequences compared to genomic DNA sequences in RAP-DB. Ok, sequence identical to either genomic DNA sequence or the predicted protein sequence based on the genomic DNA data; bs, base substitution; ins, insertion; del, deletion; ac, amino acid change.

¶ *Colletotrichum higginsianum* on the FOX hunting lines; R, resistant; S, susceptible.

** *Xanthomonas oryzae pv oryzae* on transgenic Nipponbare overexpressing the pertinent full-length rice cDNA; R, resistant, S, susceptible.

†† Yes indicates that bacterial population count was performed after inoculation of plants; data are shown in Fig. 2.

The rice cDNAs inserted in the FOX hunting lines were derived from those listed in Knowledge-based Oryza Molecular biological Encyclopedia (KOME, http://cdna01.dna.affrc.go.jp/cDNA). However, the cDNA sequences curated at the KOME site had a few errors, possibly because of errors in reverse transcription, when compared to their genomic sequences described in the Rice Annotation Project Database (RAP-DB, http://rapdb.dna.affrc.go.jp/). About 51% (18 of 35) of the cDNAs listed in Table 2 had some mutations, although most of them did not have any effect on the putative protein product.

We used blastx (http://blast.ncbi.nlm.nih.gov/Blast.cgi) to identify the closest protein homologues of those encoded by rice inserts (Table 2) in the hope of finding some of the possible underlying reasons for the observed resistance phenotype. Interestingly, most of the genes in Table 2 (or their close homologues in other species) have not been reported previously as defence-related genes.

### FOX hunting lines with zero or multiple cDNA inserts showing resistance to *Pst* DC3000

Our screening for resistance to *Pst* DC3000 identified 35 FOX lines that overexpressed single rice cDNA (Table 2). The rest of the *Pst* DC3000 resistant lines (Table 3) had no cDNA inserts (eight lines), a chimeric insert (one line), an unreadable insert (two lines), two independent inserts (four lines), two independent inserts with more than two cDNAs in one insert (one line) and more than two cDNAs at one insertion locus (eight lines).

**Table 3:** *Pst* DC3000-resistant lines with zero or more than one cDNA insert

Line No	Original line	Sequenced region	Accession no.	RAP ID	RAP description*	Response to *C. higginsianum*†
*No cDNA insert (eight lines)*						
36	K20031					S
37	K31235					S
38	K37936					S
39	R05434					S
40	R06639					S
41	R06746					S
42	K30208					S
43	K30718					S
*cDNA fragment chimera (based on full sequence) (1 line)*						
44	K02809	5′	AK071280	Os10t0110800-02	Similar to Nitrate transporter (Fragment)	R
		3′	AK071222	Os10t0539400-01	Similar to MCE-family protein MCE2C.	
*Single PCR fragment but unreadable (two lines)*						
45	K29426					S
46	R06632					S
*Two independent cDNA inserts (4 lines + 2 independent lines*‡*)*						
47	R05622					
	A	5′, 3′	AK111700	Os01t0113800-01	Protein kinase, core domain containing protein.	S
	B	5′, 3′	AK103090	Os01t0958100-02	Similar to Chloroplast SRP receptor cpFtsY precursor.	
48	K26225					
	A	5′, 3′	AK068060	Os12t0605400-01	Similar to CROC-1-like protein (Fragment).	S
	B	5′, 3′	AK065583	Os10t0438600-01	Similar to Family II lipase EXL3.	
49	R07445					
	A	5′, 3′	AK102786	None (Similar to Os05t0440250-01)	Similar to Histone deacetylase superfamily protein.	S
	B	5′, 3′	AK071245	Os03t0608800-01	PDZ/DHR/GLGF domain containing protein.	
50	K17801					
	A	5′, 3′	AK066933	Os06t0178900-01	Vacuolar H^+^-pyrophosphatase	S
	B	5′, 3′	AK101159	Os04t0528400-01	Similar to ABC transporter.	
*Two independent inserts, more than two cDNAs in one insert (one line)*						
51	K35251					
	A	5′	AK073249	Os05t0456900-01	Conserved hypothetical protein.	R
		3′	AK067396	Os01t0368700-01	Protein of unknown function DUF679 family protein.	
	B	5′, 3′	AK105874	Os01t0268800-01	Ubiquitin-associated/translation elongation factor EF1B	
*More than two cDNAs at one insertion locus (total length of cDNAs at 5′ and 3′ shorter than the observed PCR fragment) (eight lines)*						
52	K02851	5′	AK071506	Os03t0383600-01	Thiolase-like, subgroup domain containing protein.	S
		3′	AK065536	Os09t0244200-01	Conserved hypothetical protein.	
53	K37919	5′	AK073569	Os07t0175600-01	Plant lipid transfer protein and hydrophobic protein	S
		3′	AK067825	Os03t0201600-02	Similar to ischaemia/reperfusion inducible protein.	
54	K35008	5′	AK102635	Os06t0273800-01	Similar to Signal peptidase 18 subunit (Fragment).	S
		3′	AK067216	Os01t0868200-01	Zinc finger, DHHC-type domain containing protein.	
55	K36312	5′	AK072635	Os02t0532900-02	Similar to H0717B12.10 protein.	S
		3′	AK071613	Os12t0555500-01	Probenazole-inducible protein PBZ1.	
56	K41633	5′	AK072747	Os04t0657100-01	Similar to Farnesyl diphosphate synthase (Fragment).	S
		3′	AK102417	Os12t0540900-01	Similar to Tryptophanyl-tRNA synthetase	
57	R05917	5′	AK100760	Os12t0123600-01	Similar to Nucleoside-triphosphatase	S
		3′	AK069004	Os03t0220700-02	Peptidase, trypsin-like serine and cysteine domain containing protein.	
58	R05945	5′	AK065044	Os03t0749300-01	Similar to Exoglucanase precursor.	S
		3′	anti-sense of AU069314 (vector fragment)			
59	R06201	5′	AK064875	Os03t0369800-01	Similar to Novel plant SNARE 13 (AtNPSN13).	S
		3′	AK071002	Os12t0518000-01	Hypothetical conserved gene.	

* Predicted protein annotation provided by RAP-DB (http://rapdb.dna.affrc.go.jp/).

† *Colletotrichum higginsianum* on the FOX lines; R, resistant; S, susceptible.

‡ Two independent lines with two independent cDNA inserts, K18218 and K02535, are listed in Table 2.

The resistance in plants having T-DNA with no cDNA insert may be because of a disruption of a functional host gene during the transformation process with an empty vector. Therefore, it would be interesting to sequence the DNA adjacent to the T-DNA insertion site via TAIL-PCR. Multiple insertion events are expected when a pool of cDNAs is transformed en masse, via floral dip protocol, into a population of *Arabidopsis* plants. The presence of more than one cDNA at one insertion locus has been explained by Nakamura *et al.* (2007). The causatory gene for the resistance found in these lines must be determined by independent transformation to verify the function of each candidate gene.

### Resistance to *C. higginsianum*

**Figure 3:**
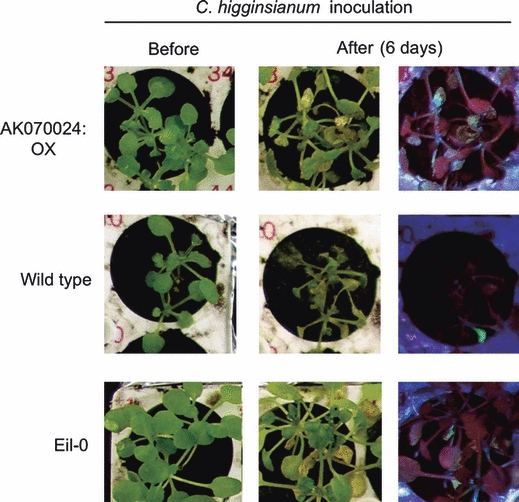
Phenotypic responses to *C. higginsianum* (2.5x10^5^ conidia/mL) dip inoculation. Three-week-old plants were used for inoculation. Compared with wild type (Col-0), AK070024:OX and Eil-0 (resistant ecotype to *C. higginsianum*) plants were still surviving 6 days after inoculation. The amounts of residual chlorophyll, which appear green under white light and red under UV, were used to assess resistance. Resistant plants appear green under white light and red under UV. AK070024:OX is the same line as that used in Fig. 1.

To determine whether the overexpression of the *RPD* genes is also effective against other types of pathogens, we tested the *RPD* lines for resistance against the fungal pathogen, *Colletotrichum higginsianum*. *Colletotrichum* species are hemibiotrophic fungi that initially feed on living tissues and continue feeding on the nutrients released from dead tissues (Perfect *et al.*, 1999). More importantly, *Colletotrichum* species produce appressoria, whose walls contain melanin, and its infection mechanism is similar to *Magnaporthe grisea*, the most important rice pathogen that causes rice blast. *C. higginsianum-*resistant lines are shown in Fig. 3. Six days after inoculation, plants of the AK070024:OX line, which was originally selected for resistance to *Pst* DC3000, survived stringent inoculation with *C. higginsianum*, whereas the wild-type plants were obviously dead (Fig. 3, photographs under white light). This indicated that AK070024:OX plants are also resistant to *C. higginsianum*. Under black light illumination (UV 365 nm), healthy tissues with intact chlorophyll exhibited red fluorescence (Eil-0 and AK070024:OX), whereas dead ones (WT) had no red fluorescence (Fig. 3, photographs under UV light). Surviving leaves of AK070024:OX and WT with severe fungal growth emitted a silvery fluorescence under UV light. In *C. higginsianum*-inoculated wild-type plants, only a part of the leaf emitted a silvery fluorescence. On the basis of these results, we used red fluorescence as a direct indicator of the health (resistance) of the inoculated tissues when it was difficult to determine whether the tissues were ‘dead or alive’ under white light.

Many of the FOX hunting lines selected for resistance to *Pst* DC3000 also showed resistance to *C. higginsianum* (Table 2 and 3). Of the 35 lines in Table 2 that were tested for *C. higginsianum* resistance, 11 (31.4%) were considered as resistant. Of the 16 lines with cDNA inserts in Table 3 that were tested for *C. higginsianum* resistance, only two were considered as resistant. All of the lines with no cDNA insert were susceptible to *C. higginsianum*.

### Transcriptomic responses of selected rice cDNAs in rice

Signal transduction plays an important role in plant–microbe interactions, where molecular signals from pathogens are perceived by specific receptors in the host, leading to a series of signalling cascades and defence responses, consequently resulting in host colonization or suppression of pathogenesis. Many genes that have been associated with plant responses to pathogen attack can be classified according to various signalling pathways mediated by signalling molecules such as salicylic acid (SA), jasmonic acid (JA), ethylene (ET) and hydrogen peroxide (H_2_O_2_). SA is a key signalling molecule involved in plant defence against biotrophic pathogens, such as the oomycete *Hyaloperonospora arabidopsidis* or hemibiotrophic pathogens, such as the *Pseudomonas syringae* bacteria (Tsuda and Katagiri, 2010)*.* SA is synthesized by plants to induce the accumulation of a set of *pathogenesis-related (PR*) genes. Benzothiadiazole (BTH) is a chemical analogue of SA that primes the SA pathway in plants (Görlach *et al.*, 1996; Lawton *et al.*, 1996). JA and ET mediate the defence signalling that is generally effective against necrotrophic pathogens such as *Alternaria brassicicola.* In plants, methyl jasmonate (MJ) and ethephon are experimentally used to activate the JA and ET pathways. The SA and JA signalling pathways generally act antagonistically (Thomma *et al.*, 1998; Glazebrook, 2005). Recently, however, it has been reported that each of the SA, JA and ET signalling sectors can positively contribute to immunity against both biotrophic and necrotrophic pathogens (Tsuda *et al.*, 2009). Reactive oxygen species (ROS), such as hydrogen peroxide (H_2_O_2_), play a major role in cellular signalling pathways and the regulation of gene expression networks in plants (Li *et al.*, 2009). The production of ROS is one of the earliest cellular responses following successful pathogen infection and elicitor treatment, leading to strengthening of the host cell walls via cross-linking of glycoproteins and activation of *PR* genes (Liu *et al.*, 2010).

To help characterize the activity of the *RPD* genes in their native genomic background, and find possible relationships between their responses to biotic and chemical stimuli, we determined the transcriptional responses of these genes to the following agents: (i) *X. oryza* pv *oryzae* (*Xoo*), (ii) *M. grisea*, (iii) MJ, (iv) ethephon, (v) hydrogen peroxide and (vi) BTH, in aseptically grown *O. sativa* ssp. *japonica* cv. Nipponbare seedlings.

**Figure 4:**
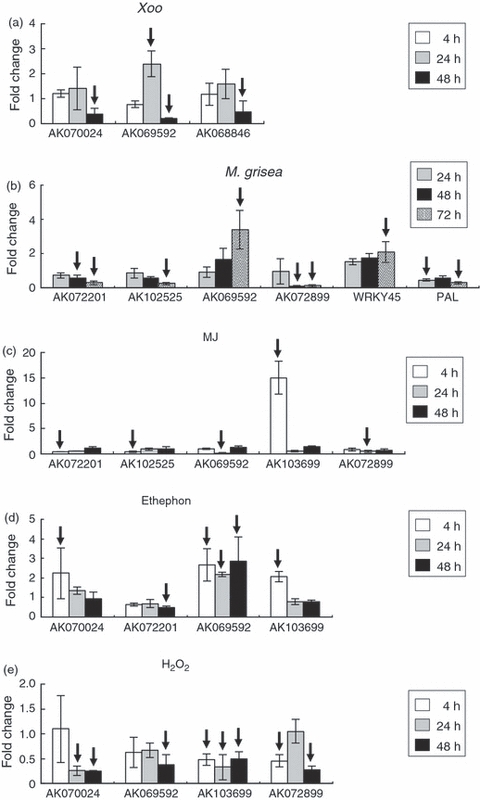
Selected rice genes that responded to biotic stress or chemical treatments in rice. Nine-day-old Nipponbare rice seedlings grown aseptically in Agripots were inoculated with *X. oryzae* pv. *oryzae* (*Xoo*) (a) or *M. grisea* (b). Compatible isolates of both pathogens were used. Other seedlings were dipped in 100 μM methyl jasmonate (MJ; c), 100 μM ethephon (d) or 10 mm H_2_O_2_ (e). Aerial parts of the plants were sampled at designated time points after treatments. Expression of 10 genes listed in the Table S1 was measured by qPCR. Additional primers for WRKY45 and phenylalanine ammonia lyase were used in the experiment using *M. grisea*. Only genes that showed clear responses (≤0.5X or ≥2X relative to control) to the treatments are shown. Arrows indicate that the fold changes are ≤0.5 or ≥2 compared to the control. Error bars indicate standard deviations (*n* = 3).

**Figure 5:**
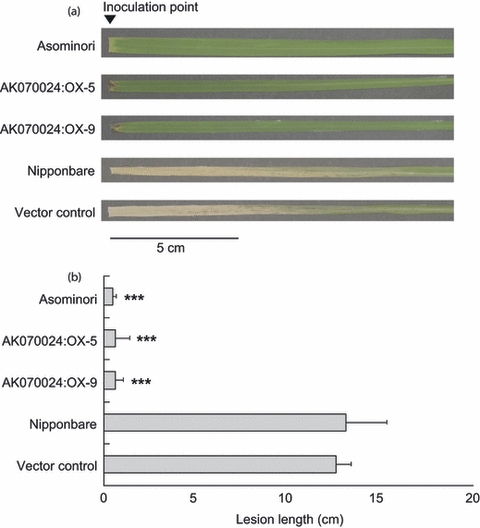
*X. oryzae* pv. *oryzae* (*Xoo*) resistance of AK070024:OX rice. Photographs (a) and lesion lengths (b) of the 6th leaf blades of AK070024:OX (T_2_), Nipponbare (WT), vector control and Asominori (*Xoo* resistant cultivar) 2 weeks after inoculation with *Xoo*. An arrowhead indicates the point of inoculation. Lesion lengths in AK070024:OX and Asominori plants were significantly lower than those in Nipponbare and vector control plants (***P < 0.001 by *t*-test). Error bars indicate standard deviations (*n* = 4–8).

Primers for qPCR amplification of *RPD* genes that were identified in ten FOX hunting lines (Lines 1–10 in Table 2) were prepared (Table S1) and used to determine their transcript levels in their native genomic background (rice). Only three of these, viz., AK070024, AK069592 and AK068846, responded to inoculation with *Xoo* with the fold changes ≤0.5 or ≥2 (Fig. 4a). These three genes were commonly suppressed at 48 h after inoculation with *Xoo*. Because some effector proteins from pathogens are known to down-regulate the expression of host defence genes (Hann and Rathjen, 2010), these three down-regulated genes may be involved in defence against pathogenic bacteria of rice. In fact, one of them, AK070024, the most promising gene identified in our screening (see the later section), conferred resistance to *Xoo* when overexpressed in rice (Table2, Fig. 5).

On the other hand, inoculation of Nipponbare with *M. grisea* elicited divergent responses. One of the selected genes (AK069592) were up-regulated, similar to WRKY45, a transcription factor that confers strong resistance to *M. grisea* when overexpressed in rice (Shimono *et al.*, 2007). The gene for phenylalanine ammonia lyase, the initial enzyme for the all-important phenylpropanoid pathway, and the other selected genes (AK072201, AK102525 and AK072899) were significantly down-regulated by *M. grisea* (Fig. 4b)*.* It is also possible that effector proteins from *M. grisea* down-regulate the expression of some rice defence genes against fungal pathogens. In fact, while the one gene up-regulated by *M. grisea* in rice failed to confer resistance to the fungal pathogen, *C. higginsianum,* in *Arabidopsis,* those that were significantly down-regulated by *M. grisea* conferred resistance to *C. higginsianum* as well as *Pst* DC3000 (Table 2).

Down-regulated transcription was also the most commonly observed response of the *RPD* genes to signalling molecules (MJ and H_2_O_2_)_._ Four of five methyl jasmonate-responsive genes and all the four H_2_O_2_-responsive genes were down-regulated (Fig. 4c,e). Meanwhile, three of four ethephon-responsive genes were up-regulated (Fig. 4d). It is likely that these genes are involved in these different defence signalling pathways. Considering the antagonism between JA and SA signalling pathways observed in many dicot plants, we examined BTH responsiveness of the four MJ down-regulated genes. However, none of them responded to BTH (data not shown). Interestingly, the most promising AK070024 was not responsive to MJ in its native genomic background, rice (data not shown). AK070024 was clearly down-regulated by H_2_O_2_ (Fig. 4e), suggesting that it may have some role in the hypersensitive response to pathogen invasion and programmed cell death (Apel and Hirt, 2004). Only one gene (AK069592) responded to (was down-regulated by) BTH (data not shown).

Many of the genes showed little response or even negative responses to pathogen infection or the defence-related signalling molecules. These results may suggest that they are involved in preformed resistance mechanism, rather than induced resistance. Alternatively, they may play indirect roles in plant defence to pathogens through ‘normal’ biological processes, such as growth, development or photosynthesis.

### Resistance to bacterial leaf blight in the transgenic rice

We examined whether *RPD* genes also extended resistance to *Xoo,* the bacterial pathogen for rice leaf blight. The cDNAs of some *RPD* genes were inserted downstream of the constitutive maize *ubiquitin* promoter, and the constructs were used to generate transgenic rice lines. Screening was performed by inoculating *Xoo* by cut-dip method at the T_1_ generation (plants from seeds of plants regenerated from transgenic calli). Overexpression of inserted cDNAs was confirmed in T_0_ plants (plants regenerated from transgenic calli) by qPCR, and T_1_ seeds derived from them were used for screening. Table 2 shows that, of eight transgenic lines tested so far, only one has shown strong resistance under our screening conditions. The detailed resistance phenotype using T_2_ plants is shown in Fig. 5. While Nipponbare (wild type) and the vector control plants developed extended lesions from the cut (inoculated) end of the leaf, AK070024:OX plants showed restricted lesion development similar to the resistant control, cv. Asominori (Fig. 5a). Lesion lengths in inoculated AK070024:OX and Asominori were about 1 cm long, whereas those in Nipponbare and vector control were about 13 cm long (Fig. 5b). These results indicate that AK070024 cDNA selected for *Pst* DC3000 resistance in *Arabidopsis* also conferred strong *Xoo* resistance in transgenic rice. Interestingly, lesions in AK070024:OX showed a dark brown colour that is likely associated with cell death (Fig. 5a).

### Resistance to rice blast in AK070024:OX rice

**Figure 6:**
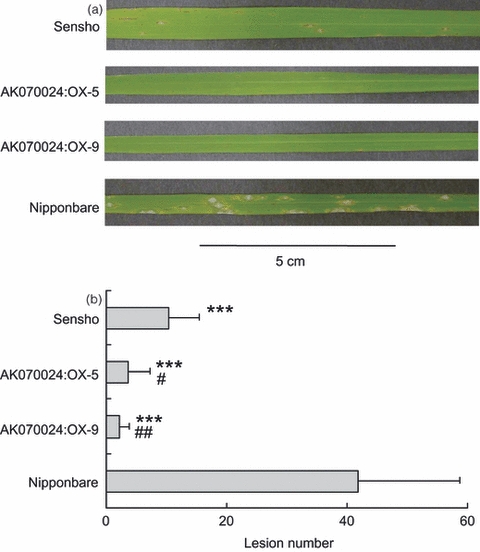
*M. grisea* resistance of AK070024:OX rice. (a) Photographs of 4th leaf blade. (b) Number of compatible lesions. Nipponbare (WT), Sensho and AK070024:OX (T_2_) plants were grown until 4-leaf stage and spray-inoculated with compatible *M. grisea*. Lesion numbers in AK070024:OX and Sensho plants were significantly lower than those in Nipponbare plants (***P < 0.001 by *t*-test). In addition, lesion number in AK070024:OX-5 and AK070024:OX-9 plants were significantly lower than those in the resistant cv Sensho (^##^*P* < 0.01 and ^#^*P* < 0.05). Error bars indicate standard deviations (*n*= 6–8).

In *Arabidopsis*, overexpression of AK070024 also conferred resistance to the fungal pathogen *C. higginsianum* (Fig. 3). Therefore, we investigated the resistance of AK070024:OX rice lines to the rice fungal pathogen *M. grisea* in comparison with Nipponbare (wild type) and the highly *M. grisea-*resistant cultivar, Sensho (Fig. 6). Compatible isolate of *M. grisea* was inoculated by spraying. Vector control plants showed *M. grisea* susceptibility similar to Nipponbare (data not shown). Lesion numbers in AK070024:OX plants were markedly lower than those in Nipponbare plants, and even less than those in Sensho, which has a strong non-race-specific resistance to *M. grisea* associated with *pi21* (Fukuoka *et al.*, 2009). Thus, overexpression of AK070024 cDNA conferred resistance to the major bacterial and fungal pathogens in both *Arabidopsis* and rice. We designated AK070024 (Os09g0533600) gene as *BROAD-SPECTRUM RESISTANCE 1* (*BSR1*) accordingly. So far, we have not observed any notable growth defect or morphological changes both in *Arabidopsis* and in rice plants overexpressing *BSR1*, except that rice plants overexpressing *BSR1* displayed a decreased germination rate.

### *BSR1* (AK070024) encodes a putative receptor-like cytoplasmic kinase

*BSR1* (AK070024) codes for a functionally uncharacterized protein of 406 amino acid residues similar to Avr9/Cf-9-induced kinase 1 according to the Rice Annotation Project Database (RAP-DB) description (Table 2). It belongs to a family of receptor-like cytoplasmic kinases (RLCKs) and was previously named as OsRLCK278 according to the phylogenetic analyses of 187 OsRLCKs (Vij *et al.*, 2008). RLCKs are a subgroup of receptor-like kinases (RLKs) that do not contain an extracellular domain or transmembrane domain but share a common monophyletic origin with RLKs (Shiu and Bleecker, 2001). BSR1 (OsRLCK278) is classified into the RLCK-VIIb subfamily. *Arabidopsis* RLCKs closest to BSR1 are At5g47070 and At4g17660 according to the phylogenic analyses by Shiu *et al.* (2004). To our knowledge, no gene in the RLCK-VIIb subfamily has been characterized; however, some genes in RLCK-VIIa, the closest subfamily of RLCK-VIIb, have been characterized. NAK (At5g02290) is a novel *Arabidopsis* protein kinase (Moran and Walker, 1993), for which no putative function nor patterns of expression have been described so far. REFSEQ (NCBI) reports that NAK has two conserved domains; viz., STKc, the catalytic domain of serine/threonine protein kinases, and PTKc, the catalytic domain of the protein tyrosine kinase (PTK) family. *Arabidopsis* BIK1 (At2g39660) and tomato TPK1b are involved in plant defence against necrotrophic fungal pathogens (Veronese *et al.*, 2006; Abuqamar *et al.*, 2008). PTO and PBS1 are well-characterized RLCKs involved in race-specific resistance to bacterial pathogens in tomato and *Arabidopsis*, respectively (Martin *et al.*, 1993; Swiderski and Innes, 2001). Sequence alignments and phylogenic tree for BSR1 and these representative RLCKs are shown in Fig. S2.

## Discussion

### Advantages of the rice-FOX *Arabidopsis* system

In this study, we screened 20 000 of rice-FOX *Arabidopsis* lines for resistance to *Pst* DC3000 infection and obtained 72 resistant lines. More than ten lines also showed resistance to the fungal pathogen *C. higginsianum*. One of the selected genes, *BSR1,* encoding a RLCK family protein, conferred remarkable resistance to both bacterial and fungal pathogens when overexpressed in *Arabidopsis* and rice. Thus, this screening system allowed us to identify at least one potentially very useful gene that can confer multiple or broad-spectrum disease resistance to both dicot and monocot plants. Further characterization of the remaining candidate genes may identify more genes of scientific and practical importance.

The rice-FOX *Arabidopsis* system has several characteristic features as a resource for screening gene function in rice. Rice is widely used as a model plant of monocots; however, the lifespan of rice is much longer than that of *Arabidopsis*, which is one of the disadvantages for using rice. In addition, large-scale functional genomics using transgenic rice is constrained by space limitations especially in countries where experimental transgenic rice need to be grown under isolated or glasshouse conditions. The rice-FOX *Arabidopsis* system is a system that is able to overcome these disadvantages. The small size and short lifespan of *Arabidopsis* enable high-speed and large-scale screening, and this is especially useful for the screening of disease resistance genes that involve complicated pathogen infection mechanisms.

Plant phenotypes resulting from the overexpression of genes do not necessarily reflect the inherent functions of the genes. It is generally accepted that such neomorphic phenotypes frequently occur when regulatory genes, such as those for protein kinases and transcription factors, are overexpressed. Because of this problem, the phenotypes in the rice-FOX *Arabidopsis* lines should be interpreted carefully. However, this issue seems to be less serious when the FOX lines are used for the screening of genes that potentially improve crops, because useful phenotype, but not the elucidation of gene functions, is the final goal in this case.

Another factor that should be considered is that the overexpression of a gene of foreign origin can yield phenotypes different from those resulting from the overexpression of the corresponding endogenous gene. This is because proteins, even if they have the same catalytic activity, may undergo different regulation in their respective genomic backgrounds. For example, the tobacco *aspartate kinase* (*AK*) gene is regulated by feedback inhibition; however, its counterpart gene in *E. coli* does not undergo regulation when overexpressed in tobacco. This enabled enhanced accumulation of free methionine in transgenic tobacco seeds expressing *E. coli AK* gene (Karchi *et al.*, 1993).

In addition, the overexpression of genes in their native host sometimes induces gene silencing, which hampers the screening of gene function. In this regard, overexpression in a foreign genomic background is less likely to induce gene silencing because of lower sequence homology with corresponding endogenous genes.

For these reasons, the FOX lines offer a unique opportunity to find previously unknown functions of rice genes. In fact, we identified a number of genes that enhance plant resistance to disease, most of which (including their orthologs in other species) have not previously been associated with resistance to any disease.

### Relationships between *Pseudomonas, Arabidopsis* and foreign rice genes

Many reports on the resistance to *Pseudomonas syringae* in *Arabidopsis* concentrated on genes involved in recognition and regulation (Katagiri *et al.*, 2002). Establishment of recognition and interaction between gene products of two interacting organisms presumably require a significant period of co-evolution, probably measured by millions of years, during which both host and pathogen evolve new gene products by random mutations. In the case of the compatible (pathogenic) relationship between *Pst* DC3000 and *A. thaliana*‘Columbia’, evolution has enabled the pathogen to overcome host defences when conditions are optimized for colonization. Our screening strategy is based on the principle of optimized pathogenesis: grow the plants in humid conditions and infect at high inoculum density to enable pathogenesis to run its full course (i.e., kill the wild-type plant). Under these conditions, only the most resistant transgenic lines can survive. Therefore, some of the genes that had been introduced into the surviving lines may be potential major disruptors of the basic host–pathogen relationship. The *RPD* genes may be explained in the context of an evolutionary arms race, where the overexpressed rice cDNA represent major mutations (‘weapons’), some of which are potent enough to overcome the screening conditions that had been tilted in favour of the compatible pathogen. These rice genes could be sufficiently novel (in the *Arabidopsis* genome) to disturb the attack and colonization machinery of *Pst* DC3000.

### Broad-spectrum disease resistance

We found that 11 of 35 single cDNA inserts identified by the *Pst* DC3000 screen also provided resistance to *C. higginsianum* (Tables 2). Broad-spectrum resistance against 2 or more different pathogens is an agronomically desirable trait. Overexpression of *Arabidopsis NPR1* (non-expressor of PR genes), a transcriptional cofactor involved in the SA pathway, conferred broad-spectrum disease resistance to *Arabidopsis*, tomato, rice, carrot and cotton (Cao *et al.*, 1998; Lin *et al.*, 2004; Quilis *et al.*, 2008; Wally *et al.*, 2009; Parkhi *et al.*, 2010). However, constitutive expression of *NPR1* also rendered plants susceptible to viral infection and hypersensitive to abiotic stresses (salt and drought) in rice (Quilis *et al.*, 2008). Zheng *et al.* (2006) reported that ectopic overexpression of WRKY33 in *Arabidopsis* made the plants resistant to necrotrophic fungal pathogens *Botrytis cinerea* and *Alternaria brassicicola* but more susceptible to *Pseudomonas syringae*. In rice, overexpression of *OsWRKY13* enhanced resistance to bacterial blight and fungal blast (Qiu *et al.*, 2007). Similarly, overexpression of the BHT-inducible *OsWRKY45* enhanced resistance not only to rice blast fungus (Shimono *et al.*, 2007) but also to bacterial blight (Tao *et al.*, 2009). Recently, it was reported that the expression of a pathogen-associated molecular pattern (PAMP) receptor of *Arabidopsis*, EFR, conferred broad-spectrum bacterial resistance in *Nicotiana benthamiana* and tomato (Lacombe *et al.*, 2010). Thus, most of the genes heretofore reported as providing broad-spectrum disease resistance are associated with signal transduction pathways. Many of the genes that conferred resistance to both *Pst* DC3000 and *C. higginsianum* in Table 2 appear to encode signalling components from their annotations. They are likely to be involved in one or more signal transduction pathways, considering their transcriptional responses to signalling molecules (Fig. 4c–e). *Arabidopsis* genes corresponding to these rice genes for putative signalling components may be those involved in defence signalling pathways that are generally effective against particular types of pathogens. This speculation needs to be verified by silencing of the endogenous genes or similar approaches.

Of eight transgenic rice lines tested, only one showed high resistance to *Xoo* (Table 2, Fig. 5) and *M. grisea* (Fig. 6), although more resistant lines are expected to be identified when we finish screening the rest of the rice lines. The *BSR1* gene is notable because it conferred broad-spectrum disease resistance in *Arabidopsis* (*Pst* DC3000 and *C. higginsianum*) and in rice (*Xoo* and *M. grisea*). To our knowledge, no other monocot gene has been reported to confer disease resistance in both monocot and dicot to both bacterial and fungal pathogens, respectively. Therefore, BSR1 may have evolved as a primal regulatory gene for pathogen resistance before the monocots split from the dicots several million years ago.

### The rarity of resistance to *Xoo* compared to *C. higginsianum*

The rarity (1/8) of resistance to *Xoo* among the transgenic rice lines contrasts with the higher portion (11/35) of cDNAs showing resistance to *C. higginsianum* in the *RPD* genes (Table 2). We surmise that the reason may lie in the different co-evolutionary backgrounds of the hosts and the corresponding pathogens. In the case of *Arabidopsis*, overexpression of the foreign rice gene represents a major mutation in both quantitative (high transcript level) and qualitative (foreign gene) terms. Such mutations may have critical effects on the *Arabidopsis*–*Pst* DC3000 or *Arabidopsis*–*C. higginsianum* interaction if they corresponded to a ‘weak link’ in the defensive arsenal of the host plant or produced novel products that confound the attack and colonization machinery of those pathogens. Mere overexpression of seven of the eight genes (see Table 2) in Nipponbare did not lead to resistance to *Xoo*; this can only indicate that these genes may not be part of the ‘weak links’ in the defence mechanisms that the rice plant evolved to deal with an invasion by *Xoo*. In other words, *Xoo* would have already evolved measures to counter the effects of most of these rice genes during the evolution of the rice–*Xoo* pathosystem. In fact, inoculation of wild-type Nipponbare with *Xoo* (or *M. grisea*) led to transcriptional repression of some of these genes (Fig. 4).

### Possible function of BSR1 in plant defence mechanism

*BSR1* encodes a putative RLCK, OsRLCK278, which belongs to the same protein family as PBS1, PTO and BIK1, the well-characterized RLCKs involved in plant defence (Fig. S2). Tomato PTO and *Arabidopsis* PBS1 are involved in race-specific resistance to *Pseudomonas syringae*. PTO recognizes a *Pseudomonas* effector protein AvrPtoB and inactivates its E3 ligase activity via phosphorylation to induce *Pst* resistance (Ntoukakis *et al.*, 2009). PBS1 is complexed with a resistance protein RPS5 and becomes cleaved by AvrPphB, a *Pst* effector with protease activity, leading to the activation of RPS5 and induction of programmed cell death in host cells (Ade *et al.*, 2007). In both cases, the RLCKs play direct roles in the recognition of bacterial effectors. *Arabidopsis BIK1* was originally isolated as a gene involved in the defence to necrotrophic fungal pathogens (Veronese *et al.*, 2006). Recently, it was reported that BIK1 associates with a flagellin receptor complex (FLS2/BAK1) to initiate plant innate immunity. BIK1 becomes rapidly autophosphorylated upon perception of flagellin, depending on both FLS2 and BAK1, and BIK1 in turn phosphorylates BAK1 and FLS2. In addition, BIK1 is also phosphorylated by another PAMP, translation elongation factor (EF-Tu). These results demonstrate that BIK1 mediates PAMPs-triggered immunity (PTI) signal transduction from multiple PAMP receptor complexes (Lu *et al.*, 2010). Interestingly, it has been shown that PBS1 and PBS1-like kinases also contribute to PTI defences and play some roles in signal integration from multiple surface-localized receptors in plants lacking *RPS5* (Zhang *et al.*, 2010).

Thus, plant RLCKs play important roles in direct or indirect recognition of pathogen-derived molecules and subsequent signal transductions. It is most likely that BSR1 protein, like BIK1 and PBS1-like kinases, functions in linking multiple PAMP receptor complexes to downstream intracellular signalling, considering the broad-spectrum disease resistance by the overexpression of *BSR1* observed in both *Arabidopsis* and rice.

## Experimental procedures

### *Pst* DC3000 culture

*Pst* DC3000 was obtained from Dr. B. J. Staskawicz (UC Berkeley, USA). All *Pst* DC3000 cultures were started from stocks containing 50% glycerol and 50% KB medium (King *et al.*, 1954) stored at −80 °C. One hundred millilitres of KB medium with 50 μg/mL of rifampicin (WAKO Pure Chemicals, Osaka, Japan) was inoculated with 0.5 mL of glycerol stock and then cultured for 16–18 h (until OD_600_∼1) in a rotary shaker set at 180 rpm and kept at 28 °C in the dark. The bacterial cells were harvested by centrifugation and resuspended at a concentration of 0.5 to 2 × 10^8^ cfu/mL in an inoculation medium consisting of 10 mm MgCl_2_ and 0.05% Silwet L-77(Lehle Seeds, Round Rock, TX).

### *Arabidopsis* culture and *Pst* DC3000 screening protocol

*Arabidopsis thaliana* ecotype Columbia (Col-0) was used as the wild type (negative control), whereas the positive control was *cpr5-2* (a gift from Dr. B.N. Kunkel, Washington University, USA), a mutant line showing very high resistance to *Pst* DC3000. The rice-FOX *Arabidopsis* lines (Kondou *et al.*, 2009) were sown in two replications at five seeds per well in 60-well plates containing pre-sterilized moist black peat moss (Super Mix; Sakata, Yokohama, Japan). After a 2-day cold (4 °C) exposure, the seeds were germinated and grown in aseptic condition for 3 weeks under a 9/15 h light/dark regime at 22 °C.

The plants were dipped for 30 s in a suspension containing *Pst* DC3000 at 0.5 to 2 × 10^8^ cfu/mL supplemented with 0.05% Silwet L-77 and incubated for 3 days in the dark and 3 days under light prior to evaluation of survival. Photographs of the plants were taken 6 days after inoculation under white fluorescent illumination and evaluated for recovery of green colour because of de novo chlorophyll synthesis using images in a computer screen (Fig. S1b). Screening of the candidate resistant lines was repeated at least twice for verification. During the initial stages of screening, we also took photographs under UV (365 nm) illumination as the red fluorescence of chlorophyll offered greater contrast. However, in the third screening, we opted to use higher inoculum and selected only the lines that had survived for 6 days after the dip inoculation for further screening and evaluation.

### Quantification of resistance by bacterial counts

To determine the degree of resistance, T_2_ seeds of the FOX lines were selected by growing them on half MS media with 1% sugar, B5 vitamins (0.04% myo-inositol, 0.0004% nicotinic acid, 0.0004% pyridoxine hydrochloride, 0.004% vitamin B1 hydrochloride), 0.05% MES, 10 mm hygromycin and 0.8% agar (adjusted to pH 5.7).

The negative control plants (WT Columbia) were sown in half MS media without the antibiotics. The 3-week-old seedlings were transferred to sterile 60-well plates containing moistened black peat moss and allowed to recover for another 2 weeks prior to dip inoculation with *Pst* DC3000 at 10^6^ cfu/mL as described previously. Three days after inoculation, aerial parts of 4–5 plants were harvested and weighed. Bacterial counting was performed using a procedure described by Katagiri *et al.* (2002).

### Insert identification

DNA was extracted from selected FOX lines using Qiagen DNeasy Plant mini kit (Valencia, CA) and then amplified by PCR using the following primers: GS17, 5′-GTACGTATTTTTACAACAATTACCAAC-3′, and GS18, 5′-GGATTCAATCTTAAGAAACTTTATTGC-3′. The number of inserts and their sizes were estimated by electrophoresis of the PCR products in agarose gels. The identity of each fragment was determined by sequencing the first 400–600 bp from the 5′ and 3′ ends and comparing the resultant data with those kept at the Knowledge-based Oryza Molecular biological Encyclopedia (KOME) website (http://cdna01.dna.affrc.go.jp/cDNA/; Rice Full-Length cDNA Consortium 2003).

### Generation of transgenic *Arabidopsis* for verification of resistance

For retransformation, the candidate rice full-length cDNAs identified by *Pst* DC3000 screening were obtained from the Rice Genome Resource Center (National Institute of Agrobiological Sciences, Japan) as *E. coli* plasmids, digested with *Sfi*I (Takara, Tokyo, Japan) and inserted downstream of 2 × CaMV *35S* promoter at the compatible *Sfi*I sites of the binary vector, pBIG2113SF (Ichikawa *et al.*, 2006). The engineered plasmids were subsequently introduced into *Agrobacterium* GV3101 by electroporation. Transgenic *Arabidopsis* lines were obtained by floral dip transformation (Clough and Bent, 1998). Transformed T_1_ seedlings were selected on a medium containing 1 mm KNO_3_, 10 μg/mL hygromycin and 0.8% agar (Nakazawa and Matsui, 2003). After 4 weeks, seedlings that survived and showed sufficient root development were individually transferred to pots containing black peat moss and grown at 22 °C in a 9/15 h light/dark regime prior to bolting and then transferred to 15/9 h light/dark regime at the start of flowering. Seeds from these T_1_ plants were used to verify the resistance phenotypes of the original T_2_ FOX lines.

### Test for resistance to *C. higginsianum*

We used a screening procedure almost identical to that applied for *Pst* DC3000, except that we used 0.25 to 2 × 10^6^ conidia/mL of *C. higginsianum* (MAFF305635 supplied by NIAS Genebank) and incubated the plants for 6 days under short-day (9 h) conditions. The response to infection (R, resistant, S, susceptible) was based on qualitative evaluation of residual green portions of infected leaves in comparison with a positive control, Eil-0, an *Arabidopsis* ecotype highly resistant to *C. higginsianum* (Narusaka *et al.*, 2004).

### Biotic stress and chemical treatments

To ensure a measurable and quantifiable response from the plants to the biotic stresses, we devised and used a high-throughput screening system for *Xoo* and *M. grisea* resistance to generate the samples for qPCR. Seeds of *O. sativa* ssp. *japonica* cv. Nipponbare were dehulled, surface-sterilized with a 1-min dip in 70% ethanol followed by a 30-min NaOHCl drench in 2.5% available chlorine. Twenty seeds were sown in two rows and grown in Agripots containing 1/2MS salts solidified with 1% agar for 9 days under a 14/10 h light/dark regime at 25 °C in a growth chamber. For *Xoo* inoculation, two longest leaves of each seedling were cut at about 2 cm from the tip and the cut ends were dipped in a suspension of *Xanthomonas oryzae* pv. *oryzae* (T7174) (OD_600_ = 1) for 30 s. For *M. grisea* inoculation, seedlings were dipped in a suspension of isolate Ina86-137 (race 007) conidia (3 × 10^5^/mL). Ina86-137 is a compatible isolate of *M. grisea* to Nipponbare. The Agripots were sealed with tape and incubated from 4 to 48 h (*Xoo*) or 24 to 72 h (*M. grisea*).

Chemical treatments of plants were performed using plants aseptically grown as described previously. The chemicals were applied by dipping the plants for 30 s in solutions. MJ (125 μm; Sigma-Aldrich, St. Louis, MO), ethephon (100 μm; Sigma-Aldrich) and hydrogen peroxide (10 mm; WAKO Pure Chemicals, Osaka, Japan) in 0.05% Tween 20 (Sigma-Aldrich) were used. Benzothiadiazole (BTH, Acibenzolar-S-methyl; Hayashi Pure Chemical, Osaka, Japan) was first dissolved in acetone and diluted in 0.05% Tween 20 to a final concentration of 300 μm. Appropriate control treatments were also performed using solvents only.

### qPCR

Total RNA was isolated from rice leaves using Isogen (Wako Pure Chemicals) followed by further purification with the RNeasy mini kit (Qiagen, Valencia, CA). First-strand cDNAs were synthesized from equal amounts of total RNA (2 ng/reaction) with a First-strand cDNA Synthesis Kit (Amersham Biosciences, Piscataway, NJ) in a total volume of 15 μL, as described by the manufacturer. Primers for quantitation of transcripts by real-time RT-PCR were designed with Primer 3 Plus (Untergasser *et al.*, 2007), usually targeted towards the (5′ or 3′) untranslated regions (UTR). Real-time RT-PCR was performed using the TaKaRa (Tokyo, Japan) Dice Real Time system as described by the manufacturer. The list of gene-specific primers is shown in Supplemental Table S1. Reaction mixtures contained 4.8 ng of cDNA template, 7.5 μL of Perfect Real SYBR premix Ex Taq II (Takara, Tokyo, Japan) and 6 pmol each of gene-specific primers per 15 μL reaction. They were incubated at 95 °C for 1 min, followed by 40 cycles of 95 °C for 5 s and 60 °C for 30 s. Transcript levels were normalized to an endogenous rice reference gene (AK059694) putatively coding for an ubiquitin-conjugating enzyme. The relative expression level of each gene was calculated using an expression ratio adjusted for gene-specific PCR amplification efficiencies and derived from 2^-ΔΔCt^ (Yuan *et al.*, 2006).

### Generation of transgenic rice lines overexpressing screened full-length rice cDNAs

To generate overexpression rice lines, full-length cDNAs, provided by the Rice Genome Resource Center (National Institute of Agrobiological Sciences, Japan), were cloned into the *SfiI* site between the maize *Ubiquitin* promoter and the nopaline synthase terminator in a binary vector, pRiceFOX (Nakamura *et al.*, 2007). The plasmids were introduced into rice (*O. sativa* ssp. *japonica* cv. Nipponbare) by an *Agrobacterium*-mediated procedure (Toki *et al.*, 2006).

### Screening for resistance to *Xoo* and *M. grisea* in transgenic rice lines

Transgenic rice seedlings were selected by hygromycin resistance (50 μg/mL). The selected rice plants were grown in a growth chamber until 6-7 leaf stage at 25 °C under a 16/8 h light/dark regime. The plants were inoculated with *Xoo* (T7174), and symptoms were evaluated 2 weeks after inoculation as described previously (Mori *et al.*, 2007). In this experiment, the 6th leaf blades of the tested plants were cut with scissors pre-wetted with inoculum (OD_600_ = 0.3) at about 5 cm from the tip, and the cut ends (about 1 cm from the ends) were dipped in a suspension of *Xoo* for 10 s.

For screening resistance to the blast fungus, we used isolate Kyu89-246 (MAFF101506, race 003.0) of *M. grisea,* which is slightly more virulent than isolate Ina86-137 (race 007). Kyu89-246 is also compatible to Nipponbare, and the symptom is similar to that of Ina86-137. It was grown on oatmeal agar plates (3% oatmeal, 0.5% sucrose and 1.6% bacto agar) at 25 °C in the dark for 12 days and under continuous illumination for 2 days to induce sporulation. To make the conidial inoculum (5 × 10^4^ spores/mL), the mycelia of *M. grisea* were scraped and gel surface was flooded with sterile water containing 0.01% Tween 20. The rice plants were grown in a greenhouse at 28 °C in the day and 24 °C at night until 4-leaf stage and spray-inoculated as described previously (Mori *et al.*, 2007). Evaluation of resistance was based on the total number of the compatible lesions that appeared on the 3rd and 4th leaf blades of each plant 6 days after inoculation.

### Sequence alignment and phylogenic analysis

Amino acid sequence alignments were generated by the CLUSTALX computer program (Thompson *et al.*, 1997). The phylogenic tree was constructed by the neighbour-joining method from the deduced amino acid sequences. Bootstrap mode (1000 replications) was used for estimating the confidence that could be assigned to particular nodes on the tree. The result was illustrated by NJ plot (Perrière and Gouy, 1996).

## Supporting information

**Figure S1** Screening for resistance to *Pst* DC3000 and *Colletotrichum higginsianum*.

**Figure S2** Protein sequence analyses between BSR1 and related RLCKs. Alignment (a) and phylogenic tree (b) for BSR1 and related RLCKs.

**Table S1** Primers used for qRT-PCR.
