# Supplementary material for: Screening for resistance against Pseudomonas syringae in rice-FOX Arabidopsis lines identified a putative receptor-like cytoplasmic kinase gene that confers resistance to major bacterial and fungal pathogens in Arabidopsis and rice
Source: Plant Biotechnol J. 2011 May;9(4):466–85. doi: 10.1111/j.1467-7652.2010.00568.x (PMC3118280; doi:10.1111/j.1467-7652.2010.00568.x)
Supplement: Supplementary file 1 [file pbi0009-0466-SD1.pdf]

SUPPLEMENTARY MATERIAL

Figure legends

Figure S1 Screening for resistance to *Pst* DC3000 and *Colletotrichum higginsianum*.

(a) Standard methods. *Arabidopsis* is usually inoculated with *Pst* DC3000 by dip or infiltration method. Resistance level is usually evaluated by bacterial counts or lesion size in the infected plants. *Arabidopsis* is also inoculated by with droplets containing conidia of *C. higginsianum* and resistance/susceptibility is based on the resultant lesion size. (b) High throughput screening of the FOX lines employed in this study. Steps 1. Grow the FOX lines in 60-well plates for 3 weeks in aseptic condition, 2. Dip inoculate with *Pst* DC3000 or *C. higginsianum*, 3. Incubate, 4. Record image by digital photography of reflected light (green) under white light or fluorescent light (red) under UV.

Figure S2 Protein sequence analyses between BSR1 and related RLCKs.

Alignment (a) and phylogenic tree (b) for BSR1 and related RLCKs. Protein sequence data are from rice (BSR1/AK070024), *Arabidopsis* (NAK/At5g02290, BIK1/At2g39660, and PBS1/At5g13160) and tomato (TPK1b/EU555286, and PTO/DQ019170). Black and gray backgrounds indicate identical and similar amino acids, respectively. Numbers at nodes indicate bootstrap values. The bar corresponds to 0.1 amino acid substitutions per site. VIIa VIIb and VIIc represent the subfamilies of RLCKs.

Figure S1

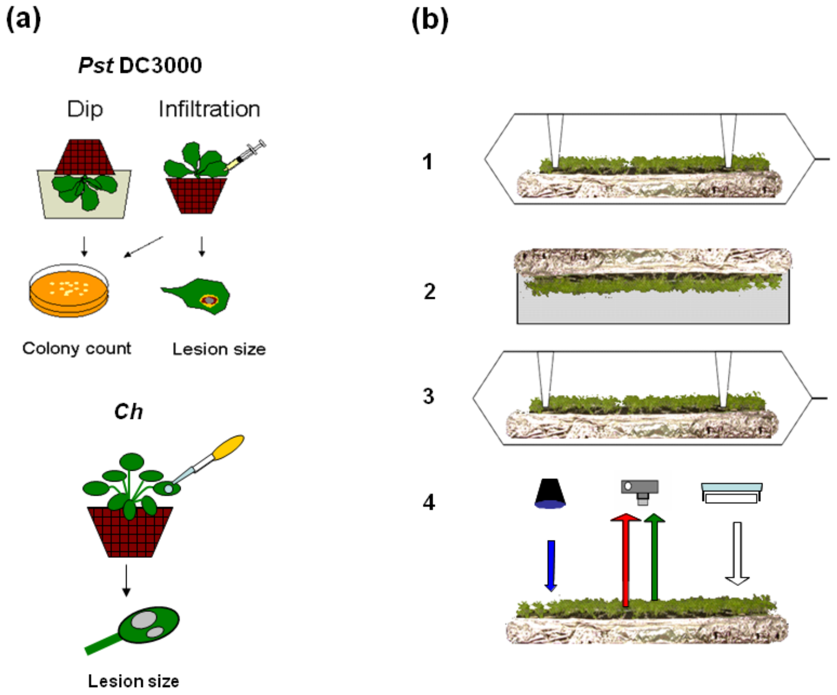

(a)
Pst DC3000
Dip
Infiltration
Colony count
Lesion size
Ch
Lesion size
(b)
1
2
3
4

Figure S2

(a)

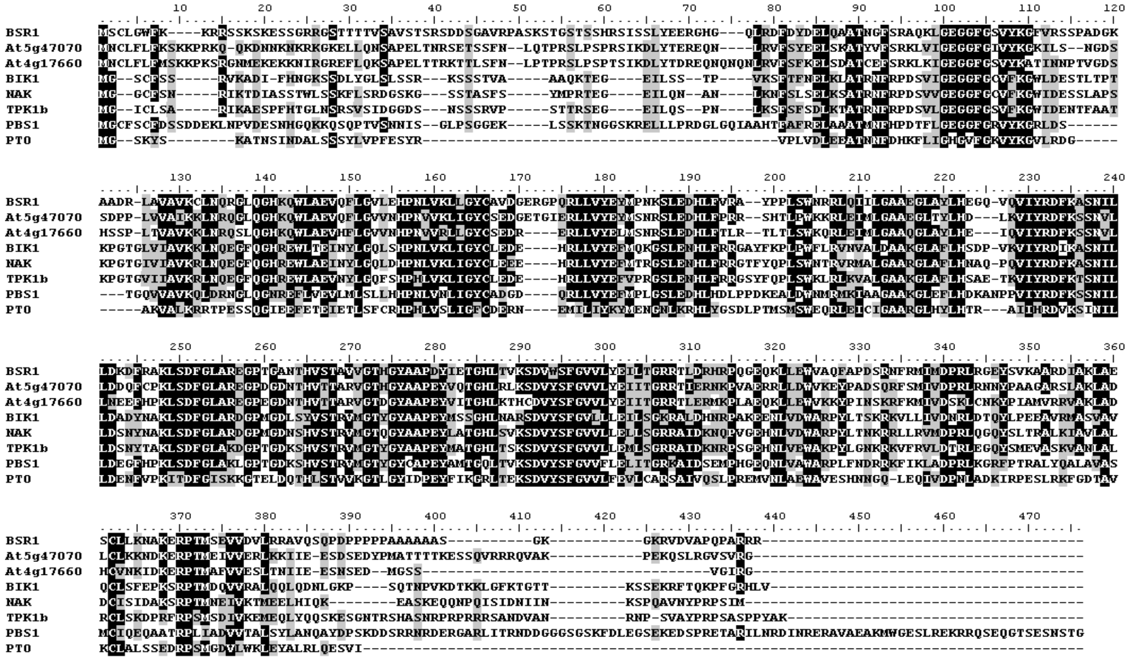
BSR1
At5g47070
At4g17660
BIK1
NAK
TPK1b
PBS1
PTO

(b)

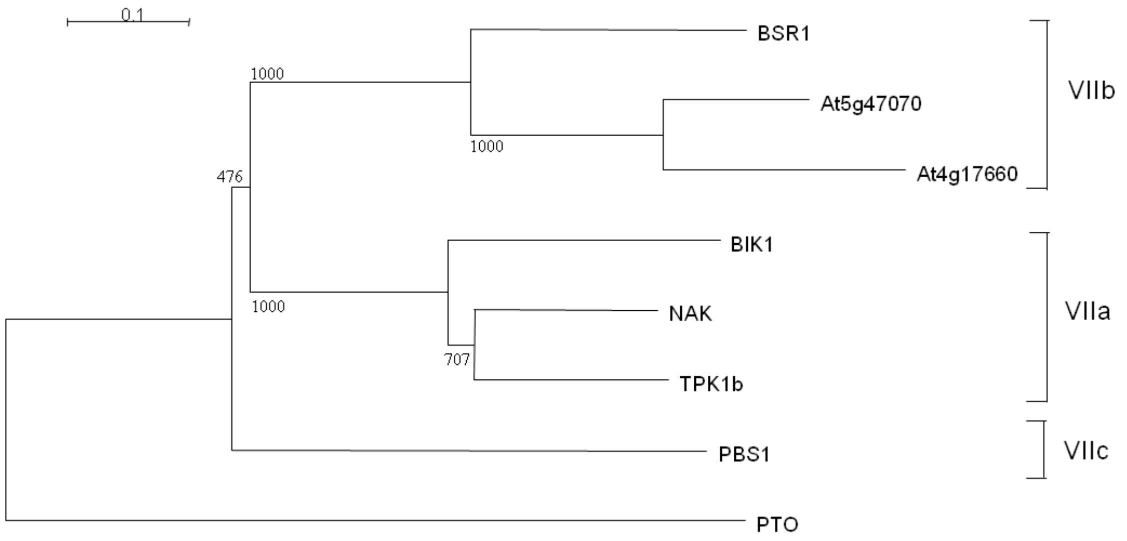
0.1
1000
1000
476
1000
707
BSR1
At5g47070
At4g17660
BIK1
NAK
TPK1b
PBS1
PTO
VIIb
VIIa
VIIc

Table S1 Primers used for qRT-PCR

Accession No.	Forward	Reverse
AK059694 (reference)	AAGGCATGGCTGATTCATGT	CAGATGTTCACATGGTGACAGTA
AK066255 (*WRKY45*)	TTCGGTGGTCGTCAAGAAC	CGTGCACAGCTGGTCGTA
AK068993 (*PAL*)	CAAAGAGAGCTCATCAGATTCC	ATGACCGTCGGTGCCAGT
AK068846	GGCTTCCTCAAGACAAAACG	TCGTCGTGTTTCACCAAAAA
AK103699	AGACCCTGCTTCGCTGGA	ATCACCATGGCGAAAAACAG
AK072201	TCACTTAATTGGCCGAAGAGA	CCTGTCAGGGCCTCACTACT
AK070024	AGGTGAGGTTGCACTCTGCT	CCAAGAATCCACCAACTCGT
AK100547	AGCTCTTGTGGGCAGTCATT	CGAATGGAAAGTAAACCTGACC
AK072899	CTGGACACCAGCCTTTTGTT	GCTTGTCTTTCTTCGGTGAATC
AK102525	ATTTTCTCTTCGCGCTTCTG	TGCATATTTTCACAGTTTTGCAC
AK102125	GCTGTTACGGCAAACGGTTA	CTCCCAAGTCCCAGTTCAAA
AK099032	TGCTTTTCTTTTTGCGCTCT	TTTGCTCAAATTGTCCCAGA
AK069592	GGCCGTTGATTGGTGTAATC	ACCGGCACATACACACAAAA
